# Pittsfield, Mass., Medical College

**Published:** 1850-03

**Authors:** 


*Pittsfield, Mass., Medical College.*—The Pittsfield Medical College building has recently been destroyed by fire, together with the Museum, etc., of the Institution. The building was insured for $2,500.

